# New Appliances and Things Medical

**Published:** 1900-05-26

**Authors:** 


### NEW APPLIANCES AND THINGS MEDICAL.

[We shall be glad to receive, at our Office, 28 & 29, Southampton Street, Strand, London, W.C., from the manufacturers, specimens of all new preparations and appliances which may be brought out from time to time.]

### HINKLITAS DISINFECTANTS.

(Hinkley and Co., 8, Ray Street, London, E.C.)

The Hinklitas disinfectants consist of a series of preparations of various forms, each designed for a special purpose, but one and all are impregnated with an antiseptic and deodorant combination, to which the name Hinklitol has been applied. The series of disinfectants includes, amongst others, Hinklitas powder for domestic and general purposes, Hinklitas pale fluid, Hinklitas red fluid, Hinklitas green oil, Hinklitas black, Hinklitas soft soap. The great value of these preparations depends on the fact that they possess in combination antiseptic as well as deodorant qualities. Antiseptics, such as carbolic acid or other tar preparations, are mainly antiseptics which possess an odour which is disagreeable rather than otherwise. The Hinklitas antiseptics contain the essential and volatile oils of the pine, which are particularly agreeable to the olfactory senses. For the ordinary purposes of disinfection, whether the end in view be the destruction of pathogenic germs or the oxidation and consequent deodorisation of poisonous and disagreeable emanation, the Hinklitas series of antiseptics constitutes a most reliable, agreeable, and safe medium.

### RONUK SANITARY POLISHES AND CLEANSERS.

(Ronuk, Limited, 83, Upper Thames Street, London, E.C.)

These preparations are in paste form, and as compared with the usual liquid varieties of polishes they present many advantages. They are possessed of a pleasant aromatic odour, and do their work effectively and rapidly. The household "Ronuk" is a sanitary polish and cleanser; it preserves and polishes wood floors in a most satisfactory manner, filling up the pores of the wood and thus preventing the accumulation of dust, as may be well seen at the National Gallery, the floors of which are regularly polished with this preparation. It may also be used for cleaning linoleum. The special black and brown "Ronuk," contained in collapsable tubes, imparts a fine polish to leather goods of all kinds, and is specially intended for the cleaning of boots. For harness there is a brilliant black waterproof polish, which is easy to use and particularly economical. The Ronuk series of polishes are certainly to be recommended both from a scientific and practical point of view.

